# Verses

**Published:** 1906-09

**Authors:** 


### VERSES.

### BLISS IN A BROOKLYN FLAT.

A folding table, bed and chair,
  A folding kitchen charms,
And last, not least, just add to that
  A pair of folding arms.

—Brooklyn Life.

---

### PULLMAN TRAGEDY.

It was a bold, bad Texas man,
Known all around as Deadshot Dan,
Who got upon a Pullman car
And journeyed from his home afar.

Next morn, when ended was the trip,
He offered but a dime for tip;
The portered black turned up his nose—
And consequently all his toes.

*New Orleans Times-Democrat.*

### THOUGHTS.

"The thoughts that germ in human minds
Are fickle as the shifting winds;
No force to drive, wireless and free
They wing their way o'er land and sea.
The glance of hate, affection's dart,
Flash eye to eye, pulse heart to heart."

*Dr. W. P. Burt.—Cosmos.*

---

### OBLIVION.

When thou art lying under ground,
Beyond the reach of sight and sound,
The world will still go round and round;
While, o'er thy dust as days go on,
Will deepen, until days are done,
The shadow of Oblivion.

*E. N. Pomeroy.*

---

### RATHER CUSS THAN PRAY.

De preacher preach till twelve er clock,
We sinners fer ter tun,
An git on solid groun an rock
Deebil ways ter shun.

De sisterin cry, an moan an pray,
Members an backsliders shout,
An call pun me ter cum away,
An hep dem Satin rout.

But de season's bad, wet an cool,
Wine-grass fills de row,
Plow-line cross er so-bock mule,
I kaint hep tink on tings below.

How kin we tink on tings on high,
An git de Hebinly way?
Dere am times wen ligion fly,
An we rudder cuss dan pray.

*B. H. Teague.*

